# Association Between Temporal Muscle Thickness and Survival in Older Patients With Brain Metastases Undergoing Radiation Therapy

**DOI:** 10.1002/cnr2.70671

**Published:** 2026-09-02

**Authors:** Lasse Pikkarainen, Tommi K. Korhonen, Antti Tolonen, Emilia K. Pesonen, Nicole Hernandez, Tanja Skyttä, Otso Arponen

**Affiliations:** ^1^ Faculty of Medicine and Health Sciences Tampere University Tampere Finland; ^2^ Department of Radiology Tampere University Hospital Tampere Finland; ^3^ Neurosurgery, OYS Neurocenter Oulu University Hospital Oulu Finland; ^4^ Research Unit of Clinical Neuroscience, Neurosurgery Oulu University Hospital and University of Oulu Oulu Finland; ^5^ Department of Oncology, Tays Cancer Centre Tampere University Hospital Tampere Finland; ^6^ Institute of Clinical Medicine, School of Medicine University of Eastern Finland Kuopio Finland

**Keywords:** brain metastasis, cancer, computed tomography, neurosurgery, radiotherapy, temporal muscle thickness

## Abstract

**Background:**

Finding reliable prognostic markers for patients with brain metastases would be beneficial since they could be used in clinical practice when making treatment decisions. Recent literature has analyzed the role of temporalis muscle thickness (TMT) as a prognostic marker in patients with brain metastases with mixed results. Previous studies have generally focused on patients younger than 65 years.

**Aims:**

The purpose of this study was to evaluate the effect of TMT measured from head CT scans on the prognosis of patients with brain metastases receiving radiotherapy aged 70 years or older.

**Methods and Results:**

We identified all patients with brain metastases treated with radiotherapy and measured their TMT from head CT scans at the date of treatment planning CT scans. The patients' demographic data, primary tumor, and treatment allocation were recorded. Sex‐specific cut‐offs for low TMT (males: 4.3 mm, females: 3.975 mm) were determined by maximizing Youden's index for 3‐month survival. We evaluated the association between TMT and 1‐, 3‐, 6‐, and 12‐month overall survival (OS) using Cox proportional hazards modelling. This retrospective study included 249 patients. The mean age of the patients in the cohort was 76.0 ± 4.9 years. Male patients had higher TMT than females (4.7 ± 1.6 vs. 3.7 ± 1.5 mm, *p* < 0.001). Altogether, 35 (14.1%), 125 (50.2%), 178 (71.5%), and 216 (86.7%) patients had deceased at 1‐month, 3‐month, 6‐month, and 12‐month points, respectively. Although having low TMT was associated with a higher risk of death at 3‐ and 6‐month timepoints (hazard ratio range: 1.41–1.54) in univariate analyses, TMT was not associated with 3‐ or 6‐month survival after adjustment for other clinical factors.

**Conclusion:**

Low TMT is not an independent marker of lower prognosis in patients aged 70 years or older with brain metastases treated with radiotherapy.

## 1 Introduction

Brain metastases are common in patients with advanced cancer. It is estimated that altogether 10%−40% of patients with solid tumors will develop brain metastases [1]. Population‐based studies suggest that the incidence of brain metastases varies between 8.3 and 14.3 per 100 000 people; the true rate is likely higher as not all brain metastases are registered [2]. The most common primary malignancies causing brain metastases are lung and breast cancers and melanoma [3]. Although the primary management options of brain metastases, that is, surgery, radiotherapy, systemic treatments in some cancers, or their combinations prolong survival [4], the median survival of patients affected by brain metastases ranges from only 2 to 21 months depending on the type of the primary malignancy [1]. Survival is particularly poor among patients aged over 65 years who, according to the Surveillance Epidemiology and End Results data, have a median survival of 4 months or less for nearly all common cancers, such as breast, esophageal, colorectal, renal, and lung cancer and melanoma [1].

Improved prognostication of patients with brain metastases at the time of diagnosis could aid in selecting the most appropriate treatment scheme for each patient: patients with better prognosis could benefit from more intensive treatments whereas those with worse survival are likely to benefit less. Hospitalization and treatments including radiotherapy and chemotherapy in the last weeks of life may negatively impact health‐related quality of life without significant benefit [5, 6, 7]. Discussing the realities of different treatment options is of utmost importance in the management of patients with poor prognosis. Accurate prognostication aids in shared decision‐making to choose between palliative care and more intensive treatments.

Recent literature has highlighted the prognostic role of muscle mass in older adults with cancer [8]. Loss of muscle mass is a characteristic of wasting syndromes cachexia and sarcopenia, and it is frequently encountered in frailty and malnutrition, all of which are associated with worse overall survival (OS) in patients with cancer. Cachexia and sarcopenia are distinct conditions even though there is much overlap between them. Cachexia is a complex “inflammatory type” of malnutrition with muscle and adipose tissue wasting, whereas sarcopenia is a simpler loss of muscle mass and strength [9, 10] Radiological evaluation of muscle mass, particularly at the level of the third lumbar vertebra (L3), strongly correlates with total lean body mass [11] and is increasingly recognized as a prognostic factor [12]. Recent literature on metastatic brain tumors [13], primary brain tumors [14], and primary central nervous system lymphomas [15] as well as other neurological and neurosurgical conditions [16, 17, 18] has supported the role of temporal muscle thickness (TMT) as a proxy of whole‐body musculature. However, the prognostic role of TMT on survival has not been studied in older patients with brain metastases.

The aim of this study was to evaluate the prognostic value of TMT among patients aged 70 years or older with brain metastases managed with radiotherapy. Understanding the prognostic role of TMT could provide new tools for prognostication and treatment allocation.

## 2 Methods

### 2.1 Study Cohort

This retrospective cohort study comprised consecutive patients aged 70 years or older who underwent brain radiotherapy, with or without surgical resection, for brain metastases at Tampere University Hospital during the years 2014–2022. Patients were identified from the local electronic radiotherapy database.

We included patients who had a histologically confirmed diagnosis of a primary malignancy and a brain lesion consistent with brain metastases on head CT or MRI scan. Patients were excluded if they lacked appropriate CT scans (e.g., CT images were not available or the slice thickness was over 3.0 mm). Surgically treated patients who received brain radiotherapy were included.

The permission to use and publish register data was granted and the need for written informed consent was waived by the Institutional Review Board of the Pirkanmaa Wellbeing Services County (study code: R22628) in accordance with the national law. As the study was retrospective, it did not require approval of the ethics committee according to the national law.

### 2.2 Radiological Measurements

**FIGURE 1:**
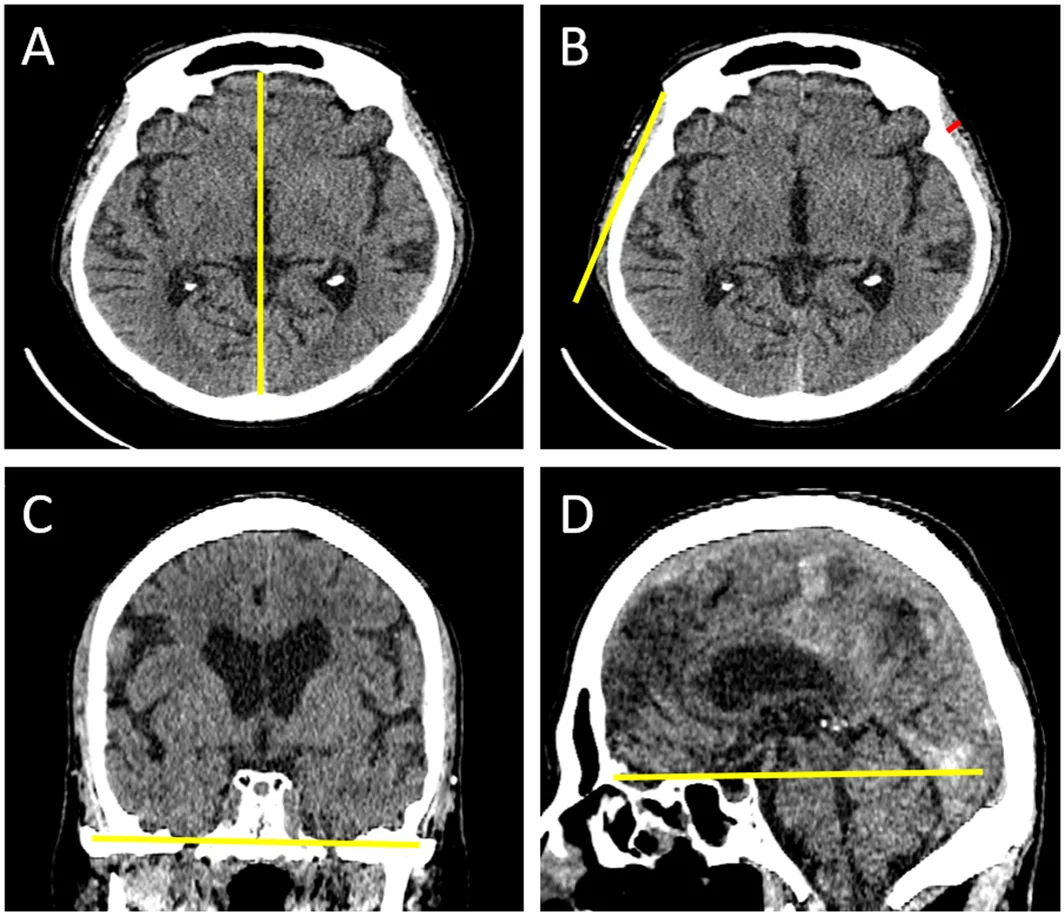
Measurement of temporal muscle thickness (TMT). The measurement plane is aligned parallel to the anterior and posterior insertions of the falx cerebri (A), the falx cerebri in the coronal plane (C), and the frontal skull base (D). Bilateral temporal muscle thicknesses (shown only on the left side of the image as a red line) are measured perpendicular to tangent lines (shown only on the right side of the image as a yellow line) (B). The average TMT from the bilateral measurements is used for statistical analysis.

One observer (L.P.) measured the TMT on the first radiotherapy planning head CT scan for each patient. If a diagnostic CT head scan was performed within a month before the first planning scan, it was used instead. The measurements were performed using Commit (RIS IDS7 Radiology Desktop, Sectra AB, Linköping, Sweden) blinded to the outcomes. Another observer (O.A.) performed the measurements in a sub‐cohort of 19 patients (38 TMT measurements). The measurement protocol was adapted from previous studies [17, 19]. Briefly, the measurement plane was oriented parallel to the cerebral falx (axial and coronal planes), and tangential to the floor of the middle cranial fossae (coronal plane) using the three multiplanar reconstruction dimensions. The measurement plane was windowed to linear width 80 and length 40 Hounsfield units. TMT measurements were performed on the first axial slice above the bony orbit. A line was drawn from the anterior insertion of the temporalis muscle tangentially to the cranium and the TMT was recorded as the thickest part of temporal muscle perpendicular to this line. Identifiable fat, fascia and vascular structures were not included in the measurement. The average of the left and right TMT (i.e., mean TMT) was used in survival analyses. The slice alignment and TMT measurements are illustrated in Figure 1.

### 2.3 Clinical Parameters

Clinical parameters were collected from the electronic patient records and included age, height, weight (recorded closest to the date of the treatment planning CT scan), sex, and type of the primary malignancy. Furthermore, we collected information on any history of brain metastasis resection prior to radiotherapy and the type of radiotherapy received, classified as either whole‐brain radiotherapy (WBRT) or stereotactic radiotherapy (SRT). SRT was defined as radiotherapy delivered with a mean dose per fraction of ≥ 5 Gy. One‐, 3‐, 6‐, and 12‐month OS were calculated from the time of the CT scan until the end of follow‐up (22 December 2023) or death.

### 2.4 Statistical Analyses

Complete case analyses were used, with no cases omitted. Categorical values are presented as absolute values and as percentages, and continuous values as means ± standard deviations (SDs) unless otherwise stated. Inter‐reader reproducibility was evaluated using the intraclass correlation coefficient (ICC) with a two‐way mixed‐effects model. The Mann–Whitney *U* test was used to test the associations between categorized TMT and continuous clinical variables (age, body mass index [BMI], and continuous TMT). The chi‐square test was used to evaluate the association between categorical values.

To optimize sex‐based cut‐off values for normal and low TMT, Youden's indexes (sensitivity + specificity − 1) were calculated for males and females for 3‐month survival, and the cut‐offs with the highest Youden's indexes were chosen. The cut‐off was optimized for 3‐month survival in accordance with previous research suggesting that patients with less than 3‐month survival are unlikely to benefit from oncological treatments [8].

We evaluated the association between TMT and 1‐, 3‐, 6‐, and 12‐month survival using the Kaplan–Meier estimation and Cox proportional hazards modeling, and report the associated hazard ratios (HRs) and 95% confidence intervals (CIs). We constructed separate Cox proportional hazards models for these survival horizons, with censoring applied at the respective time points, to evaluate each clinically relevant time horizon separately. Variables significant in univariate Cox analyses were further analyzed in multivariable models. Statistical significance was set at *p* < 0.05. SPSS for Windows (version 29.0.1.0, 1989–2020, SPSS Inc., Armonk, NY, USA) was used for statistical analyses.

## 3 Results

### 3.1 Patient Demographics

A total of 249 patients (136 males [54.6%; mean age 76.0 ± 4.9 years] and 113 females [45.4%; mean age 76.4 ± 5.3 years]) met the inclusion criteria (Table 1). Mean BMI was 24.9 ± 4.0 kg/m^2^ and 25.1 ± 5.2 kg/m^2^ for males and females, respectively. Altogether 160 (64.3%) patients had undergone a scan with a slice thickness ≤ 1 mm, 31 (12.4%) a scan with a slice thickness > 1 mm but ≤ 2 mm, and 58 (23.3%) a scan with a slice thickness > 2 mm but ≤ 3 mm. Male patients had higher TMT than females (4.7 ± 1.6 vs. 3.7 ± 1.5 mm, *p* < 0.001). The most common cancer types were lung cancer (*n* = 113; 45.4%), female breast cancer (*n* = 29, 11.6%), and melanoma (*n* = 27, 10.8%). Most patients received radiotherapy only (*n* = 226 (90.8%), of whom 151 (60.6%) received WBRT and 98 (39.4%) SRT). Twenty‐three (9.2%) patients underwent surgical resection followed by postoperative radiotherapy (7 [30.4%] patients received WBRT and 16 [69.6%] SRT). Altogether 35 (14.1%), 125 (50.2%), 178 (71.5%), and 216 (86.7%) patients had deceased at the 1‐month, 3‐month, 6‐month, and 12‐month timepoints, respectively.

**TABLE 1:** Patient characteristics.

	All patients (*N* = 249)	Normal TMT^a^ (*n* = 137 (55.0%))	Low TMT^a^ (*n* = 112 (45.0%))	*p*
Age (years), mean (SD)	76.2 ± 5.1	76.0 ± 4.9	76.4 ± 5.3	0.865
Sex, *n* (%)				< 0.001*
Female	113 (45.6)	49 (43.4)	64 (56.6)	
Male	136 (54.6)	88 (64.7)	48 (35.3)	
BMI				0.049*
25 kgm^2^	109 (43.8)	54 (39.4)	55 (49.1)	
≥ 25 kg/m^2^	110 (44.2)	69 (50.4)	41 (36.6)	
Missing	30 (12.0)	14 (10.2)	16 (14.3)	
Primary cancer, *n* (%)				0.064
Lung	113 (45.4)	53 (46.9)	60 (53.1)	
Female breast	29 (11.6)	19 (65.5)	10 (34.5)	
Melanoma	27 (10.8)	21 (77.8)	6 (22.2)	
Gastrointestinal	22 (8.8)	12 (54.5)	10 (45.5)	
Renal	16 (6.4)	10 (62.5)	6 (37.5)	
Other^b^	42 (16.9)	22 (52.4)	20 (47.6)	
Type of radiotherapy, *n* (%)				0.018*
WBRT	151 (60.6)	74 (49.0)	77 (51.0)	
SRT	98 (39.4)	63 (64.3)	35 (35.7)	
Surgical treatment, *n* (%)				0.467
No	226 (90.8)	126 (55.8)	100 (44.2)	
Yes	23 (9.2)	11 (47.8)	12 (52.2)	
TMT (mm), mean (SD) [median (mm), IQR]	4.3 ± 1.6 [4.3, 2.0]	5.4 ± 1.1 [5.2, 1.4]	2.9 ± 0.9 [3.1, 1.3]	< 0.001*

*Note:* Patient characteristics are also presented separately for patients with normal and low temporal muscle thickness according to sex‐based cut‐offs optimized for 3‐month survival.

Abbreviations: BMI, body mass index; IQR, interquartile range; SD, standard deviation; SRT, stereotactic radiotherapy; TMT, temporal muscle thickness; WBRT, whole‐brain radiotherapy.

^a^ Cut‐offs acquired by maximizing the Youden's index.

^b^ Including patients with multiple primary tumors (*n* = 13 [5.2%]).

* Significant value.

### 3.2 TMT Cut‐Offs

TMT cut‐off values acquired by maximizing the Youden's index for 3‐month survival were 4.3 mm (sensitivity 46.7%, specificity 74.6%) for males and 3.975 mm (sensitivity 64.6%, specificity 49.2%) for females. Forty‐eight (35.3%) males and 64 (56.6%) females had low TMT.

### 3.3 Inter‐Reader Reproducibility

Inter‐reader reproducibility was assessed in a randomly selected sub‐cohort of 19 (7.6%) patients (38 temporal muscle measurements). The mean TMT measurements were 4.5 ± 1.7 mm and 4.6 ± 1.7 mm for Reader 1 and 2, respectively. The ICCs were excellent for both the single (ICC 0.942 (95% CI 0.891–0.969), *p* < 0.001) and average (ICC: 0.970 (95% CI: 0.942–0.984), *p* < 0.001) measurements.

### 3.4 Survival

**FIGURE 2:**
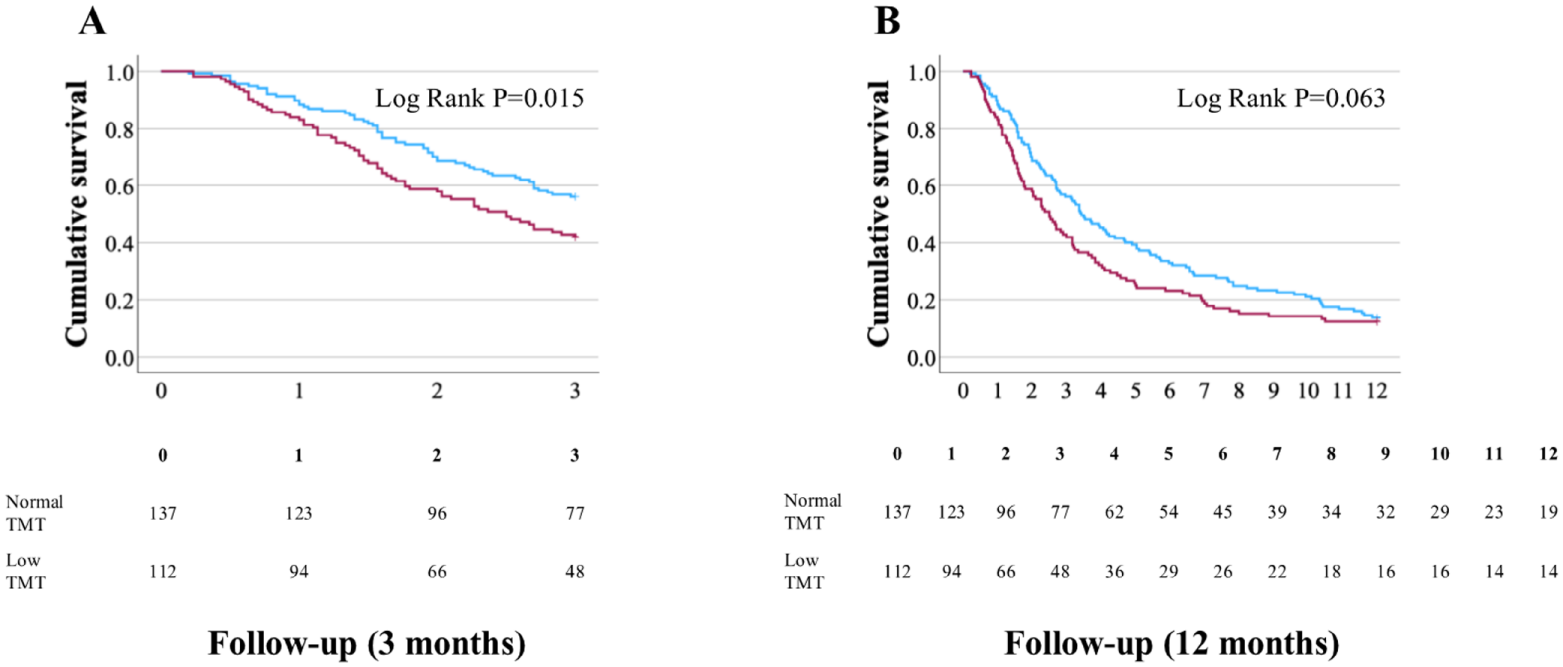
Kaplan–Meier curves for 3‐month overall survival (OS; A) and 12‐month OS (B) of patients with normal and low temporal muscle thickness (low: red line, normal: blue line) according to Youden's index‐defined cut‐off values. The number‐at‐risk tables are presented below the curves.

The median OS estimate for the whole cohort was 90 (95% CI 76.8–103.2) days, while the estimates for patients with high and low TMT were 103.0 (95% CI 81.59–124.41) days and 75.0 (59.45–90.56) days, respectively. Low TMT was not associated with 1‐month (HR 1.51 [95% CI 0.78–2.94]), and 12‐month (HR 1.29 [95% CI 0.98–1.68]; Figure 2B) survival in univariate models. However, low TMT was associated with 3‐ (HR 1.54 [95% CI 1.08–2.18]; Figure 2A), and 6‐month (HR 1.41 [95% CI: 1.05–1.89]) survival in univariate analyses, but not in multivariable models adjusted for age, BMI, surgical treatment, and radiotherapy type (Table 2).

**TABLE 2:** Univariate and multivariable Cox regression analyses for 1‐month, 3‐month, 6‐month, and 12‐month survival. Number of patients included in the analyses unless otherwise stated: 249^a^.

	1‐month survival		3‐month survival		6‐month survival		12‐month survival	
	Univariate	Multivariable^c^	Univariate	Multivariable	Univariate	Multivariable	Univariate	Multivariable
	HR (95% CI)	HR (95% CI)	HR (95% CI)	HR (95% CI)	HR (95% CI)	HR (95% CI)	HR (95% CI)	HR (95% CI)
Age (years)	0.98 (0.91–1.05)		1.02 (0.99–1.06)		1.03 (1.002–1.06)*	1.04 (1.01–1.07)*	1.03 (1.004–1.06)*	1.04 (1.01–1.07)*
Sex								
Female	1		1		1		1	1
Male	0.88 (0.45–1.71)		1.42 (0.99–2.03)		1.26 (0.94–1.70)		1.36 (1.04–1.79)*	1.27 (0.96–1.66)
BMI (kg/m^2^)^c^								
< 25 kgm^2^	1	1	1		1		1	
≥ 25 kg/m^2^	0.32 (0.14–0.76)*	0.31 (0.13–0.73)*	0.83 (0.57–1.21)		0.82 (0.60–1.13)		0.78 (0.59–1.04)	
TMT^b^								
Normal	1		1	1	1	1	1	
Low	1.51 (0.78–2.94)		1.54 (1.08–2.18)*	1.40 (0.98–2.00)	1.41 (1.05–1.89)*	1.26 (0.93–1.70)	1.29 (0.98–1.68)	
Surgical treatment								
Yes	1		1	1	1	1	1	1
No	23.55 (0.18–3076.40)		4.26 (1.57–11.53)*	3.55 (1.30–9.68)*	5.86 (2.41–14.28)*	4.26 (1.73–10.50)*	3.22 (1.83–5.66)*	2.44 (1.37–4.34)*
Type of radiotherapy								
SRT	1	1	1	1	1	1	1	1
WBRT	4.21 (1.63–10.84)*	5.98 (1.80–19.85)*	2.48 (1.66–3.70)*	2.11 (1.40–3.19)*	2.71 (1.94–3.78)*	2.53 (1.79–3.59)*	2.34 (1.75–3.12)*	2.34 (1.73–3.15)*

Abbreviations: BMI, body mass index; CI, confidence interval; HR, hazard ratio; TMT, temporal muscle thickness.

^a^ Number of events at 1‐month, 3‐month, 6‐month, and 12‐month timepoints: 35 (14.1%), 125 (50.2%), 178 (71.5%), and 216 (86.7%).

^b^ Cut‐offs acquired by maximizing Youden's index for 3‐month survival; 4.3 mm for males and 3.975 mm for females.

^c^ Number of patients with information on BMI: 219 (88.0%). Univariate and multivariable models evaluating or containing BMI were limited to 219 patients. Number of events at 1‐, 3‐, 6‐, and 12‐month timepoints among these 219 patients: 27 (12.3%), 106 (48.4%), 150 (68.5%), and 187 (85.4%).

* Significant value.

The association between TMT and survival outcomes were evaluated in patients with lung cancer (*n* = 113). Low muscle index did not associate with lower 1‐month (17 events, HR 1.00 [95% CI: 0.38–2.58]), 3‐ (63 events, HR 1.48 [95% CI: 0.90–2.46]), 6‐ (84 events, HR 1.36 [95% CI: 0.88–2.01]), or 12‐month (100 events, HR 1.30 [95% CI: 0.88–1.91]) survival. Thus, multivariable analyses were not conducted. Given that TMT was not associated with survival in the largest tumor sub‐cohort, the association between TMT and survival outcomes were not tested in smaller sub‐cohorts.

In the multivariate models, receiving WBRT instead of SRT was associated with lower (HR 5.98 [95% CI 1.80–19.85]) and higher BMI with higher (HR 0.31 [95% CI: 0.13–0.73]) 1‐month survival. Patients who received WBRT and did not undergo pre‐radiotherapy surgery had lower 3‐month, 6‐month, and 12‐month survival (Table 2). In addition to WBRT and non‐surgical treatment, age was associated with lower 6‐month and 12‐month survival. Univariate and multivariable models for 1‐month, 3‐, 6‐, and 12‐month survival are presented in Table 2.

## 4 Discussion

We evaluated the prognostic role of TMT in a cohort of older patients with brain metastases treated with radiotherapy. The survival rate of these patients is extremely poor, with a median OS of only 90 days in our cohort. Low TMT, defined with sex‐specific optimized cut‐offs, was associated with higher three‐ and six‐month OS in the univariate analyses. This association was not evident after adjusting for other clinical parameters. Regarding other variables, patients who were older, had a lower BMI, did not undergo surgical resection, and underwent WBRT instead of SRT had a higher risk of death. These results are not surprising since surgery is generally performed on younger patients with solitary brain metastases and better prognosis, whereas WBRT is preferred to SRT in patients with multiple metastases and thus poorer prognosis [20]. Our results suggest that TMT is not an independent prognostic factor in patients with brain metastases undergoing radiotherapy.

Previous studies have examined the prognostic value of TMT in patients diagnosed with brain metastases in younger patients (Table 3). Of the six previous studies evaluating the prognostic effect of low TMT, three reported that low TMT was associated with lower survival [22, 23, 24], whereas [21] did not find an association and [25, 26] did not report any associations between categorized TMT and survival after multivariable adjustment. Several factors may explain why low TMT was not associated with survival in our cohort. First, the patients in the previous studies were on average more than 10 years younger than the patients in our cohort. Second, the patients in previous studies that reported median OS had a median OS ranging from 3 to 11 months compared to our patients' median OS of 3 months. Furthermore, unlike in our study where only approximately 10% of the patients underwent surgery for the brain metastases, three of the previous studies included only surgically treated patients [21, 22, 24], while four of the previous studies included both surgically treated patients with or without radiotherapy and non‐surgically treated patients [13, 23, 25, 26].

**TABLE 3:** Summary of the previous studies evaluating temporal muscle thickness in patients with brain metastases.

References	Age	Cohort description	Modality	Outcomes	Median/mean OS (months)	Cut‐offs	Results
Current study	Mean age: 76.2 ± 5.1 years	249 older patients (≥ 70 years) undergoing brain radiotherapy for BMs.	CT	OS	3 (median)	Younden's index‐defined cut‐offs: 4.3 mm for males and 3.975 mm for females	Univariate: Low TMT was associated with lower survival at 3 (HR 1.54 [95% CI 1.08–2.18]) and 6‐month (HR 1.41 [95% CI 1.05–1.89]) timepoints. TMT was not associated with 1‐ (HR 1.51 [95% CI 0.78–2.94]) and 12‐month (HR 1.29 [0.98–1.68]) OS. Multivariable: TMT was not associated with the risk of death after the adjustment for clinical variables (see Table 2).
Klingenschmid et al. [21]	Mean age: 60 years (95% CI 58.8–62.1 years)	199 patients undergoing surgery for 1–3 BMs (106 [53.3%] males, 93 [46.7%] females)	MRI (T1‐weighted MR images)	OS	31.3 (mean)	Younden's index‐defined cut‐offs: 5.45 mm for OS > 6 months, 6 mm for OS > 12 months, and 5.83 mm for OS > 24 months	Univariate: TMT was not associated with OS (HR not reported). Multivariable: Not reported.
Cho et al. [22]	Median age: 63 years (range 27–87 years)	566 patients with BMs from NSCLC treated with Gamma Knife radiosurgery (269 [48%] males, 297 [52%] females)	MRI (T1‐weighted MR images)	OS, LPFS	Not reported	Existing cut‐offs: 6.3 mm for males and 5.2 mm for females	Univariate: Low TMT associated with the risk of death (HR 1.908 [95% CI 1.550–2.349]). Multivariable: Low muscle mass was associated with higher risk of death (HR 1.680 [95% CI 1.347–2.095]) after the adjustment of age groups, sex, number of BMs, presence of extra‐cranial metastases, NSCLC subtypes, Karnofsky Performance Status groups, recursive partitioning analysis classes, and concomitant immunotherapy or targeted therapy. No significant differences in the mean LPFS between patients with normal and low TMT.
Kim et al. [23]	Median age not reported for the whole cohort. Median ages of patients with normal and low TMT were 61.7 years (range 33–90 years) and 66.9 years (range 36–90 years)	222 patients with BMs from NSCLC (133 [59.9%] males, 89 [40.1%] females)	MRI (T1‐weighted MR images)	OS	5 (median)	Median‐based cut‐offs: 7.7 mm for males and 7.0 mm for females	Univariate: Normal TMT associated with lower risk of death (HR 0.71 [95% CI 0.54–0.93]). Multivariable: Normal TMT associated with lower risk of death (HR 0.73 [95% CI 0.55–0.96]) after the adjustment for sex, age group, and ECOG status.
Ilic et al. [24]	Median age: 64 years (interquartile range 58–70 years)	141 patients with BMs from NSCLC treated with surgery (72 [51%] males, 69 (49%) females)	MRI (T1‐weighted MR images)	OS	11 (median)	Median‐based cut‐off: 11 mm	Univariate: Not reported. Multivariable: Low TMT associated with higher risk of death (HR 1.7 [95% CI 1.2–2.6]) after the adjustment for age group, modified frailty index, and the presence of multiple BMs.
Yeşil Çinkir et al. and Çolakoğlu Er [25]	Median age: 60 years (range 39–78)	94 patients with BMs from lung cancer (71 [75.5%] NSCLC and 23 [24.5%] SCLC; 82 [87.2%] males, 12 [12.8%] females)	MRI (T1‐weighted MR images)	OS	3 (median)	Median‐based cut‐off: 4.32 mm	Univariate: Normal TMT associated with lower risk of death (HR 0.812 [95% CI 0.66–0.99]). Multivariable: TMT was not associated with the risk of death after the adjustment for nutritional indexes (HR not reported).
Furtner et al. [26]	Median age: 60 years (range: 23–88 years)	146 patients with BMs from melanoma (96 [65.8%] males, 50 [34.2%] females)	MRI (T1‐weighted MR images)	OS	7.6 (median)	Median‐based cut‐offs, values not reported	Univariate: Normal TMT associated with lower risk of death (HR 0.721 [95% CI 0.642–0.810]). Multivariable: Not reported for categorical data.
Furtner et al. [13]	BC cohort Median age: 54 years, range 30–85 years NSCLC cohort Median age: 62 years (range 33–88 years)	435 patients from two cohorts comprised of 188 patients with BMs from female BC and 247 patients with BMs from NSCLC (139 [56.3%] males, 108 [43.7%] females)	MRI (T1‐weighted MR images)	OS	Not reported	Median‐based cut‐offs: 5.4 mm for patients with BC, 5.9 mm for patients with NSCLC	BC cohort Univariate: Not reported for categorical data. Multivariable: Not reported for categorical data. NSCLC cohort Univariate: Not reported for categorical data. Multivariable: Not reported for categorical data.

Abbreviations: BC, breast cancer; BM, brain metastases; CI, confidence interval; CT, computed tomography; ECOG, Eastern Cooperative Oncology Group; HR, hazard ratio; LPFS, local progression‐free survival; MR, magnetic resonance; MRI, magnetic resonance imaging; NSCLC, non‐small‐cell lung cancer; OS, overall survival; SCLC, small‐cell lung cancer; TMT, temporal muscle thickness.

We defined sex‐based cut‐offs by maximizing the Youden's index for 3‐month survival. Notably, our cut‐offs were lower than those of other researchers, except for [25] (Table 3). Lower cut‐offs for normal and low TMTs in our cohort reflect the older population compared to previous research [13, 21, 22, 23, 24, 25, 26]. We determined cut‐offs for this cohort because other studies based their cut‐offs on younger patients. Our cut‐offs may be affected by the fact that most patients with breast cancer and melanoma had high TMT. Interestingly, although males are known to have thicker temporal muscles than females, only two previous studies [21, 23], in addition to the current one, defined cut‐offs for males and females separately.

Ours is the first study on the prognostic role of TMT in older patients with brain metastases treated with radiation therapy. Our study had a consecutive and relatively large patient sample. However, our study also had some limitations. Firstly, only a minority of our patients underwent surgery before radiotherapy, causing low power in this subgroup. Future studies on older patients with surgically treated brain metastases are warranted. Secondly, high TMT was overrepresented in some cancers. This could affect the OS results by making the prognosis partly dependent on the primary tumor. Thirdly, we derived sex‐specific TMT cut‐offs. Although this approach is common in the literature, it may lead to overfitting and external replication of the results is encouraged. Fourthly, due to the retrospective nature of this study, functional, nutritional, or frailty measures had not been recorded. Lastly, we did not adjust the models for performance status, intracranial tumor status (number of lesions or intracranial tumor volume), extracranial disease status, primary tumor control, and systemic therapy. Studies with larger sample sizes are needed to account for these variables and the potential multicollinearity arising from the inclusion of these highly correlated factors.

Although low TMT, defined with sex‐specific optimized cut‐offs, was associated with higher 3‐ and 6‐month OS in univariate analyses, TMT was not an independent prognostic factor in patients aged 70 years or older diagnosed with brain metastases and treated with radiotherapy. TMT should not be regarded as a prognostic factor in patients aged 70 years or older diagnosed with brain metastases and treated with radiotherapy.

## Author Contributions

**Lasse Pikkarainen:** conceptualization, methodology, investigation, formal analysis, visualization, resources, writing – original draft, writing – review and editing, data curation. **Tommi K. Korhonen:** conceptualization, methodology, data curation, investigation, validation, formal analysis, visualization, resources, writing – review and editing. **Antti Tolonen:** conceptualization, methodology, data curation, investigation, visualization, resources, writing – review and editing. **Emilia K. Pesonen:** conceptualization, methodology, data curation, investigation, visualization, resources, writing – review and editing. **Nicole Hernandez:** conceptualization, methodology, data curation, investigation, visualization, resources, writing – review and editing. **Tanja Skyttä:** conceptualization, methodology, data curation, investigation, visualization, resources, writing – review and editing. **Otso Arponen:** conceptualization, methodology, data curation, investigation, validation, formal analysis, supervision, funding acquisition, visualization, project administration, resources, writing – review and editing.

## Funding

This work was funded by the Finnish Medical Foundation, Cancer Foundation Finland, Cancer Society of Pirkanmaa, and Orion Research Foundation. The funders had no role in the design of this study, its execution, analyses, interpretation of the data, or the decision to publish the results.

## Ethics Statement

The permission to use and publish register data was granted and the need for written informed consent was waived by the Institutional Review Board of the Pirkanmaa Wellbeing Services County (study code: R22628) in accordance with the national law. As the study was retrospective, it did not require approval of the ethics committee according to the national law.

## Conflicts of Interest

The authors declare no conflicts of interest.

## Data Availability

All relevant data are included in the manuscript.
